# Machine learning approach to predict postoperative opioid requirements in ambulatory surgery patients

**DOI:** 10.1371/journal.pone.0236833

**Published:** 2020-07-31

**Authors:** Akira A. Nair, Mihir A. Velagapudi, Jonathan A. Lang, Lakshmana Behara, Ravitheja Venigandla, Nishant Velagapudi, Christine T. Fong, Mayumi Horibe, John D. Lang, Bala G. Nair

**Affiliations:** 1 Lakeside High School, Seattle, WA, United States of America; 2 Department of Electrical Engineering and Computer Sciences, University of California, Berkeley, CA, United States of America; 3 Haverford College, Haverford, PA, United States of America; 4 Perimatics LLC, Bellevue, WA, United States of America; 5 Department of Anesthesiology and Pain Medicine, University of Washington, Seattle, WA, United States of America; 6 Department of Anesthesiology, VA Puget Sound Hospital, Seattle, WA, United States of America; Cleveland Clinic, UNITED STATES

## Abstract

Opioids play a critical role in acute postoperative pain management. Our objective was to develop machine learning models to predict postoperative opioid requirements in patients undergoing ambulatory surgery. To develop the models, we used a perioperative dataset of 13,700 patients (≥ 18 years) undergoing ambulatory surgery between the years 2016–2018. The data, comprising of patient, procedure and provider factors that could influence postoperative pain and opioid requirements, was randomly split into training (80%) and validation (20%) datasets. Machine learning models of different classes were developed to predict categorized levels of postoperative opioid requirements using the training dataset and then evaluated on the validation dataset. Prediction accuracy was used to differentiate model performances. The five types of models that were developed returned the following accuracies at two different stages of surgery: 1) Prior to surgery—Multinomial Logistic Regression: 71%, Naïve Bayes: 67%, Neural Network: 30%, Random Forest: 72%, Extreme Gradient Boost: 71% and 2) End of surgery—Multinomial Logistic Regression: 71%, Naïve Bayes: 63%, Neural Network: 32%, Random Forest: 72%, Extreme Gradient Boost: 70%. Analyzing the sensitivities of the best performing Random Forest model showed that the lower opioid requirements are predicted with better accuracy (89%) as compared with higher opioid requirements (43%). Feature importance (% relative importance) of model predictions showed that the type of procedure (15.4%), medical history (12.9%) and procedure duration (12.0%) were the top three features contributing to model predictions. Overall, the contribution of patient and procedure features towards model predictions were 65% and 35% respectively. Machine learning models could be used to predict postoperative opioid requirements in ambulatory surgery patients and could potentially assist in better management of their postoperative acute pain.

## 1. Introduction

Pain is a commonly reported symptom among patients after surgery [1,2]. However, the management of acute postoperative pain continues to be difficult for both the patients and the health care providers. Patients with unrelieved postoperative pain are associated with slower recovery, delayed ambulation and increased risks of infection and thromboembolism [3]. Further, patients with poorly controlled postoperative pain are at higher risk of developing chronic pain [4]. In addition to patient impact, there are also deleterious consequences of inadequate pain management for hospitals, including extended length of stay, increased risk of readmission, and increased cost of care [3].

Opioids are often used to manage postoperative pain [5]. Despite their widespread use to mitigate pain, opioid use is also associated with negative side effects including neurological effects, respiratory depression, gastrointestinal effects, and pruritus [6]. For these reason, opioid-sparing multimodal analgesic options are increasingly being adopted for optimal pain control in the perioperative setting [7]. Nevertheless, opioids still have a critical role in acute postoperative pain management especially for procedures where a primary neuraxial, regional or local infiltration is not possible.

Predicting postoperative pain levels and opioid requirements could facilitate proactive strategies that can optimize pain control to avoid underuse or overuse of opioids. Towards this, previous studies have retrospectively looked for predictors of postoperative pain and analgesic consumption, identifying four significant predictors including age, type of surgery, anxiety levels, and psychological distress [8]. Some of these studies have focused on specific types of surgeries and patient population to determine factors associated with postoperative opioid usage [9,10]. To date, a review of the published literature indicates the lack of rigorous research pertaining to the identification of perioperative predictive factors for acute postoperative pain and opioid requirements across a wide spectrum of surgeries. Furthermore, previous studies used traditional statistical methods as opposed to trying machine learning, potentially limiting their predictive abilities [11].

Recently, artificial intelligence methods such as machine learning have increasingly been used in the medical field to predict clinical events [12]. Machine learning is particularly suited to analyze large datasets, compute complex interactions, identify hidden patterns, and generate actionable predictions in clinical settings. In many cases, machine learning has been shown to be superior to traditional statistical techniques [13–18]. Machine learning models offer a promising method to predict pain levels and opioid requirements following surgery. However, the applications of machine learning in the context of opioids have been specific and limited in scope, with attempts to predict opioid overdose risk among Medicare beneficiaries with opioid prescriptions and inadequate pain management in patients suffering from depression as two examples [19,20].

In this study, we developed machine learning models to predict postoperative opioid requirements for a wide range of outpatient surgeries. The models were developed and validated using a large dataset comprised of patient, procedure and provider data. The models were built to predict postoperative opioid requirements prior to surgery using preoperative data and at the end of surgery using both preoperative and intraoperative data.

## 2. Materials and methods

### Study setting

This study was approved by the University of Washington Institutional Review Board (IRB# STUDY00002256). Requirement for patient consent was waived. Our academic medical center performs approximately 18,000 adult surgical procedures annually with ambulatory surgeries comprising approximately 40–45% of the surgical volume.

### Data sources

**Fig 1:**
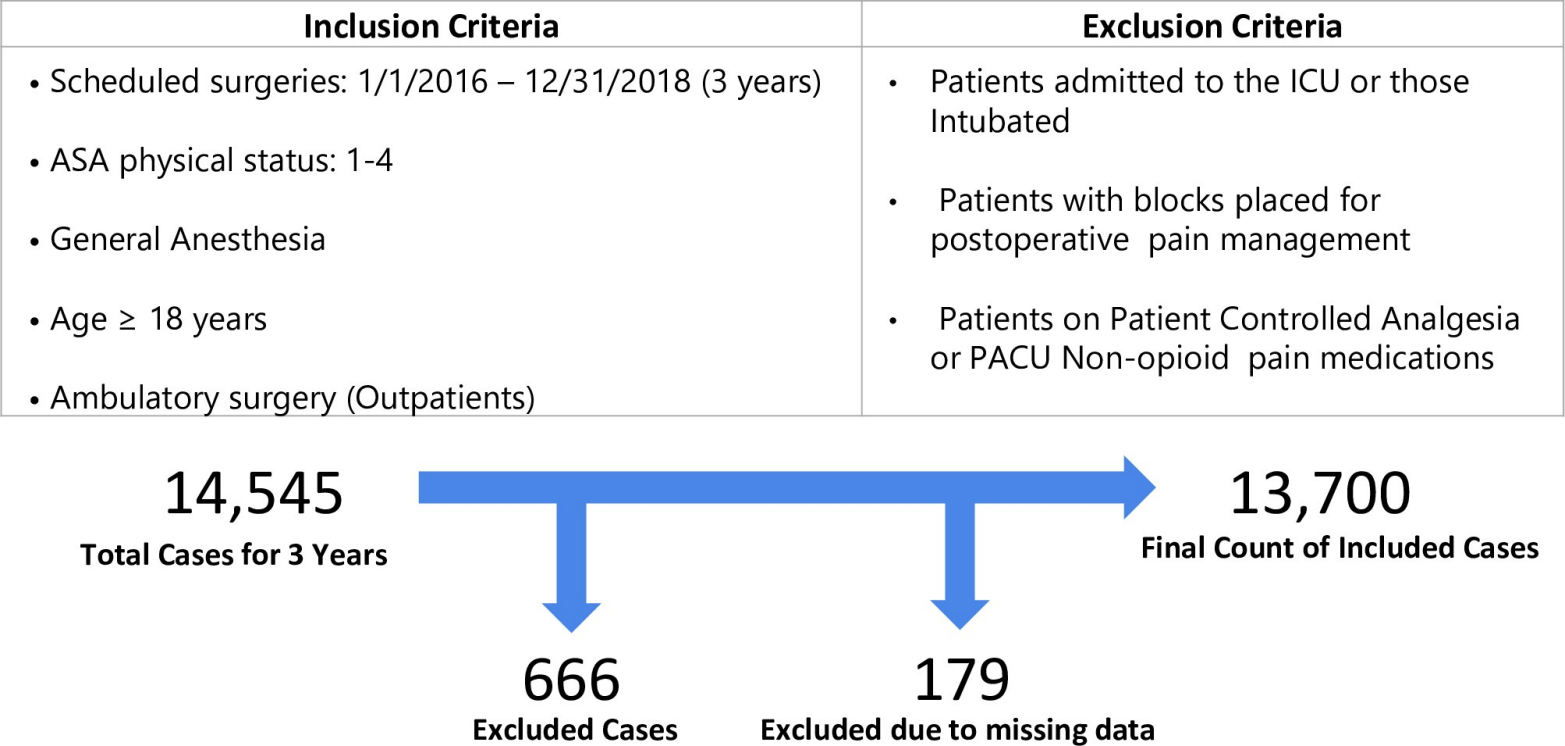
Inclusion and exclusion criteria used to select the cohort for the study are shown. The total count of patients, the number of exclusions and the final count of patients are shown.

Patient and procedure information on ambulatory surgeries for 3 years (2016–2018) were extracted from our institution’s perioperative information management system data warehouse. Only adult (≥ 18 years of age) outpatients that received general anesthesia were included. Patients who were on patient-controlled analgesics or who received non-opioid analgesics in the post anesthesia care unit (PACU) were excluded. Additionally, patients who remained intubated, or had a peripheral nerve block placed for postoperative pain management were also excluded. The inclusion and exclusion criteria as well as the patient counts are presented in Fig 1. The choice of data variables for model development was largely based on prior literature that identified factors influencing postoperative pain levels or opioid consumption [8,21–24]. Patient and procedure specific parameters prior to and during surgery were considered to achieve the goal of developing models to predict postoperative opioid requirements both prior to and at the end of surgery. The summarized list of patient and procedure specific variables used for model development is outlined in Table 1.

**Table 1:** Parameters used in prediction models. The parameters marked in green were used for postoperative opioid requirements prediction prior to surgery and parameters marked in blue were added to the prediction models to perform a postoperative opioid requirements prediction at the end of surgery.

Patient specific parameters	Procedure specific parameters
Age	Surgical specialty
Gender	Procedure type (scheduled)
Body Mass index (BMI)	Procedure duration (estimated)
Race	Preoperative holding area opioids
ASA physical status	Preoperative holding area other drugs (Acetaminophen, Gabapentin, Celecoxib)
Medical history or anomalies	Preoperative pain levels
• Cardiac	Anesthesia method
• Pulmonary	• Endotracheal general anesthesia
• Renal	• Laryngeal mask airway
• Endocrine (diabetes)	• Total intravenous anesthesia
• Musculoskeletal	Inhalation agents (type and duration)
• Hepatic	Intraoperative opioids (MME)
• Neurological	Other intraoperative meds
• Cancer	• Acetaminophen
• Sleep apnea (Diagnosed or at risk)	• Ketamine
• Chronic pain	• Ketorolac
Social history	• Naloxone
• Smoking status	• Esmolol infusion
• Alcohol abuse	• Lidocaine infusion
• Drug abuse	• Propofol infusion
Psychiatric/Neurological issues	Patient position
• Anxiety	Local infiltration
• Depression	Input fluids (Crystalloids, colloids, blood)
• Post-traumatic stress disorder (PTSD)	Output fluids (Urine, blood loss, gastric output)
• Spinal cord injury	
Home medications	Providers
• On opioids	• Surgeon
• On non-opioid pain medications	• Anesthesiologist

### Data preparation

Several data preparation steps were performed prior to model development. Records with outlier data values and key missing data points were identified. They comprised only a small fraction of the total number of records (<1%, N = 179). Hence, they were simply excluded from model development.

Data elements relating to medical, social and psychiatric histories, were embedded in free text fields in the electronic medical record (EMR). Standard natural language processing techniques were used to generate modelling features from these free text data. Pain levels recorded in the EMR were a combination of patient reported numeric rating (0 –no pain to 10 most severe pain) or nurse assessed pain levels (none, mild, moderate and severe pain). For modeling purposes, numeric rating pain scores were normalized to pain levels (0: no pain, 1–3: mild pain, 4–6 moderate pain and 7–10 severe pain) [25].

Administered opioids could be of different types and potencies. Therefore, Morphine Milligram Equivalent (MME) representation was used to consolidate the opioid doses into a single normalized value [26–28]. The MME conversion ratios used for the study are tabulated in S1 Table. Numeric MME values are less practical to interpret and act in a clinical setting than categories of opioid requirements that correspond to pain levels. Hence, we proceeded to categorize the MME values into four compartments of opioid requirement–None/very low, low, medium and high. This categorization was based on the average MME opioids used when the pain levels were none, mild, moderate and severe. The mean MME requirements for no, mild, moderate and severe pain levels are shown in Table 2. The average value of MME requirements for adjacent pain levels were used to determine the MME ranges of opioid requirement categories. MME ranges for the opioid requirement categories were: None/very low (0–3 MME), low (3–11 MME), medium (11–25 MME), and high (≥25 MME). The categorized postoperative opioid MME requirement served as the dependent variable for the predictive models.

**Table 2:** Mean postoperative MME requirements for different peak pain levels in PACU.

Peak Pain in PACU	Mean Postoperative MME	MME ranges for opioid requirement categories
No pain (Pain score = 0)	1	None/very low: 0–3
Mild (Pain score 1–3)	5	Low: 3–11
Moderate (Pain score 4–6)	17	Medium: 11–25
Severe (Pain score 7–10)	34	High: > 25

Procedure types proved to be useful in defining decision boundaries, but were too numerous for practical use without further processing. Hence, they were thus aggregated into a smaller list based on the location of surgical site and whether the procedure was open or closed. The recategorized procedure types and counts are in S2 Table.

### Model development

The master dataset was randomly split into two groups comprised of a training dataset (80% of the records) and a “holdout” dataset (20% of the records) for unbiased validation. Model development and parameter tuning were each performed using the training dataset. Five models of different classes were developed to predict postoperative opioid requirements: Multinomial Regression, Naïve Bayesian, Neural Network, Random Forest and Extreme Gradient Boosting Trees. Models were developed to predict probabilities of postoperative opioid requirements for each procedure among the four categories (none/very low, low, medium and high). Models were trained to predict opioid requirements prior to surgery using only preoperative data and then a second time at the end of surgery using both preoperative and intraoperative data. This two-phase model development matched the expected user requirements: an initial estimate prior to surgery and a more informed estimate post-surgery both having utility in the clinical setting.

Model development was performed in R programming environment (R Foundation for Statistical Computing, Vienna, Austria) [29].

### Model validation

**Fig 2:**
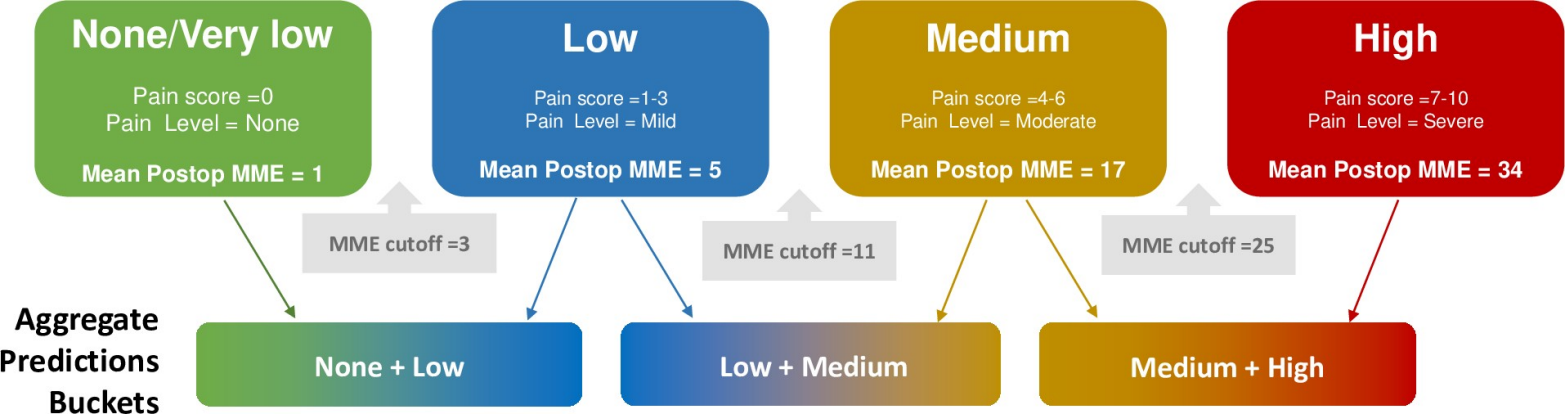
The concept of using aggregate model prediction for model validation is shown. The models predicted probabilities of postoperative opioid requirements in four buckets None/very low, Low, Medium and High. For model validation, the ability to predict within two adjacent buckets (None + Low, Low + Medium, Medium + High) was considered. The aggregate prediction bucket that had the highest combined prediction probability was considered as the model prediction. The predicted aggregate bucket was compared against the bucket corresponding to the actual opioid requirement for estimating the model accuracy.

Model validation was performed on the “holdout” dataset that was not used for training. Prediction accuracy over the holdout set was used to differentiate models. The output for model predictions were probabilities in each of the established four categories: none, low, medium, or high opioid requirement. Prediction accuracy could be computed based on the single category with the highest prediction probability and comparing that against the category in which the actual opioid requirement falls. However, this approach becomes overly strict especially when the model predicted probabilities in adjacent categories are similar. In consultation with our clinical partners we adopted an alternate, more balanced, approach to compute accuracy. Instead of simply selecting the category with the highest prediction probability the models were evaluated based on their ability to perform an aggregate prediction within two adjacent opioid requirement categories: None + Low, Low + Medium and Medium+ High. The aggregate prediction bucket with the highest combined prediction probability was determined to be the model prediction. The predicted aggregate bucket was compared to the bucket corresponding to the actual opioid requirement for estimating the model accuracy. The concept is shown in Fig 2. The model with the highest accuracy was chosen and further evaluated in terms of precision and recall. Further, prediction accuracies for different surgical specialties were also determined. To explain the model predictions in terms of feature importance, we used permutation method. The method works by randomly shuffling data one feature at a time for the entire dataset and calculating how much the prediction accuracy decreases when a feature is excluded. A larger change in prediction accuracy represents a greater importance of that feature.

## 3. Results

A total of 13,700 patients were included in the study. The patient and procedure characteristics are presented in Table 3. The mean ± SD age of the patients was 51±17 years with geriatric patients (>65 years of age) comprising 22% of the population. The mean BMI was 28.4 ± 7.2 with 35% of the patients obese (BMI > 30). Female patients were a higher fraction (58%) while racial demographics was predominantly white (83%). A significant portion of the patients suffered from depression (24%) or anxiety (26%). Among home medications, 52% of the patient cohort were on non-opioid pain medications and 23% on opioids. Chronic pain was diagnosed in 3.8% of the patients. The mean ± SD of the procedure duration was 75±56 minutes with the main surgical specialties being General (22%), ENT (18%) and Urology (15%). Table 3 also presents the characteristics of the training and testing data subsets. The patient and procedure factors were well matched between the two data subsets with none the factors significantly different between the datasets.

**Table 3:** Primary patient and procedure characteristics observed in the overall (N = 13,700), training (N = 10,960) and testing (N = 2740) datasets. Proportions are presented for categorical variables while mean ± standard deviation (SD) are shown for continuous variables. The comparison of characteristics between the training and testing datasets is also presented.

	Overall (N = 13,700)		Train (N = 10,960)		Test (N = 2740)		diff	
Characteristics	Counts	Proportions /Mean ± SD	Counts	Proportions /Mean ± SD	Counts	Proportions /Mean ± SD	p-value	
Age (years)		51 ± 17		52 ± 17		51 ± 17	0.43	
• Geriatric (Age≥65y)	3,352	24%	2,683	24%	669	24%		
Sex:								
• Male	5,699	42%	4,556	42%	1,143	42%		
• Female	8,001	58%	6,404	58%	1,597	58%	0.91	
Race:								
• White	11,355	83%	9,062	83%	2,293	84%	0.22	
• African American	640	5%	527	5%	113	4%	0.14	
• Asian	1,040	7%	845	8%	195	7%	0.31	
• Other	665	5%	526	5%	139	5%	0.58	
BMI (kg/m^2^)		28.4 ± 7.2		28.4 ± 7.2		28.5 ± 7.2	0.67	
• Obese (BMI>30)	4,094	30%	3,259	30%	835	30%		
ASA physical status								
• ASA ≥ 3	4,740	35%	3,791	35%	949	35%	0.98	
Medical history or anomalies								
• Cardiac	5487	40%	4,393	40%	1094	40%	0.90	
• Pulmonary	3,647	27%	2,907	27%	740	27%	0.63	
• Renal	1,978	14%	1,609	15%	369	13%	0.11	
• Endocrine (diabetes)	1,624	12%	1,313	12%	311	11%	0.38	
• Musculoskeletal	6,874	50%	5,536	51%	1,338	49%	0.12	
• Hepatic	647	5%	511	5%	136	5%	0.54	
• Neurological	6,088	44%	4,872	44%	1,216	44%	0.96	
• Cancer	4,902	36%	3,898	36%	1,004	37%	0.30	
• Sleep apnea (diagnosed or at risk)	6,470	47%	5,198	47%	1,272	46%	0.36	
• Chronic pain	516	4%	409	4%	107	4%	0.71	
Social history								
• Smoking status	1,303	10%	1,038	9%	265	10%	0.78	
• Alcohol abuse	1,692	12%	1,355	12%	337	12%	0.95	
• Drug abuse	1,310	10%	1,023	9%	287	10%	0.08	
Psychiatric/Neurological issues								
• Anxiety	3,521	26%	2,781	25%	740	27%	0.08	
• Depression	3,342	24%	2,642	24%	700	26%	0.12	
• Post-traumatic stress disorder (PTSD)	326	2.4%	253	2.3%	73	2.7%	0.31	
• Spinal cord injury	255	1.9%	204	1.9%	51	1.9%	1.00	
Home medications								
• On opioids	3,107	23%	2,463	22%	644	24%	0.26	
• On non-opioid pain medications	7,116	52%	5,682	52%	1,434	52%	0.66	
Surgical specialty								
• General	2,955	22%	2,352	21%	603	22%	0.55	
• Neurological	676	5%	544	5%	132	5%	0.79	
• Orthopedic	1,091	8%	872	8%	219	8%	0.98	
• Gynecology	1,436	10%	1,147	10%	289	11%	0.93	
• ENT	2,511	18%	2,016	18%	495	18%	0.71	
• Urology	1,999	15%	1,606	15%	393	14%	0.70	
• Thoracic	504	4%	408	4%	96	4%	0.63	
• Vascular	167	1%	140	1%	27	1%	0.25	
• Plastic	1,619	12%	1,302	12%	317	12%	0.68	
• Oral	400	3%	306	3%	94	3%	0.09	
Surgery duration (min)		75 ± 56		75 ± 56		75 ± 55		0.86

BMI–Body Mass Index, ASA–American Society of Anesthesiologists, ENT–Ear Nose Throat.

### Exploratory data analysis

**Fig 3:**
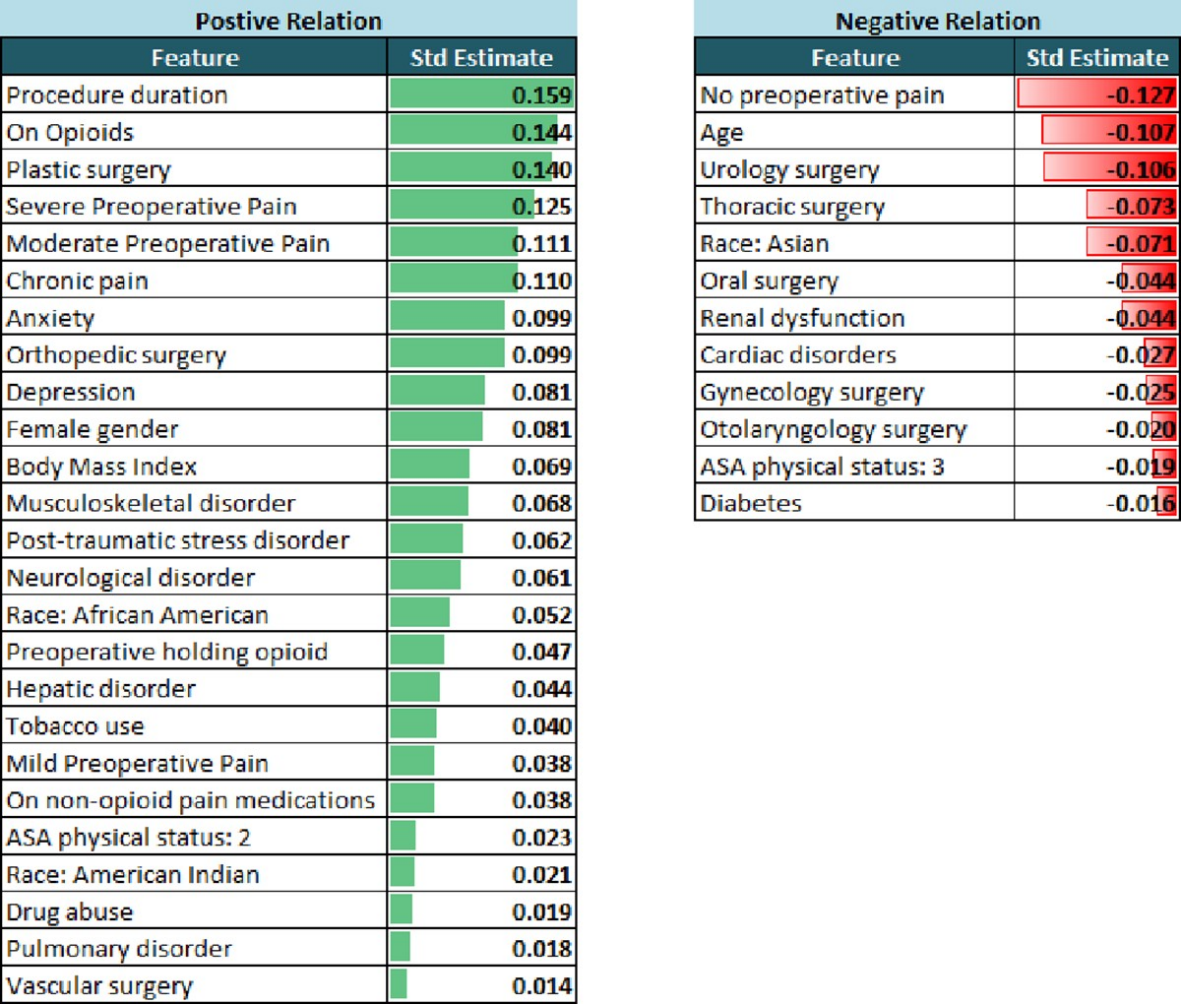
Bivariate relationships between preoperative features and postoperative MME opioid requirements are shown. Only statistically significant (p < 0.05) relations are shown with standard estimate (Std Estimate) representing the strength of the relationship. The positive and negative relations are show in green and red colors respectively.

Exploratory data analysis was performed to understand relationships between variables and to inform modeling steps. To have a basic understanding of the factors affecting opioid requirements, bivariate relationships between patient or procedure factors and postoperative opioid requirements were found. The statistically significant preoperative factors are shown in Fig 3. Longer duration procedures, patients on opioids and plastic surgeries were top factors that were related to higher opioid requirements. On the other hand, no preoperative pain, older age, urological surgeries were the top factors related to lower opioid requirements.

### Model predictions

Models were validated on a hold-out dataset (N = 2740, 20% of total data). The prediction accuracies for all five models when including just the preoperative features and when adding intraoperative features are show in Table 4. Random Forest and Multinomial Regression models had the best accuracy. Adding the intraoperative features did not enhance the prediction accuracy of the models significantly. Table 5 presents detailed results for the best performing model, which was the Random Forest. Model accuracies for different surgical specialties are shown. Model accuracies varied for different surgical specialties. Oral and thoracic surgeries had the highest accuracies, though counts of these surgeries were comparatively small. General and plastic surgeries had lower accuracies. Since the model accuracies when predicting opioid requirements at the beginning and end of surgery were similar further analyses focused on the beginning of surgery stage. Table 6 presents the recall (sensitivity) and precision (positive predictive value) of random forest model predictions. Recall was highest when the model predicted to the “none+low” aggregate category. For the “low+medium” category recall was very poor. Precision was highest for “none+low” and “medium+high” categories while lowest for “low+medium”. Table 7 shows the confusion matrices for each category of opioid requirement with true positive, true negative, false positive and false negative counts and rates. Overall, the model performance was poorer when predicting higher opioid requirements. The model had most difficulty predicting the “low+medium” opioid requirement as compared with the other categories. Model performance metrics in predicting single categories is also presented in S3 and S4 Tables.

**Table 4:** Prediction accuracies of different models prior to surgery and at the end of surgery are presented. The models utilized preoperative factors for predictions prior to surgery while the end of surgery predictions used both preoperative and intraoperative factors.

Validation Data Set: N = 2740				
Observed Opioid Requirements in Validation Data Set				
None	Low	Medium	High	
1,290 (47%)	409 (15%)	536 (20%)	505 (18%)	
Model		**Accuracy**		
		**Prior to Surgery**		**End of Surgery**
Multinomial Logistic Regression		**71%**		**71%**
Naïve Bayes		**67%**		**63%**
Neural Network		**30%**		**32%**
Random Forest		**72%**		**72%**
Extreme Gradient Boost		**71%**		**70%**

**Table 5:** Detailed prediction accuracies of random forest model for different categories of surgeries and aggregate opioid requirements. Prediction accuracies prior to and after surgery are shown.

Surgical Specialty	Mean opioid requirement (MME)	Accuracy	
		Beginning of surgery	End of surgery
**General (N = 603)**	12.1 ± 17.8	**67%**	**70%**
**Gynecology (N = 289)**	11.0 ± 17.2	**71%**	**72%**
**Neuro (N = 132)**	12.6 ± 25.1	**70%**	**74%**
**Oral (N = 94)**	7.7 ± 17.1	**87%**	**86%**
**Orthopedic (N = 219)**	19.7 ± 21.2	**74%**	**70%**
**Otolaryngology (N = 495)**	11.3 ± 25.4	**68%**	**67%**
**Plastic (N = 317)**	20.0 ± 23.4	**67%**	**66%**
**Thoracic (N = 96)**	2.9 ± 8.4	**95%**	**94%**
**Urology (N = 393)**	6.8 ± 14.4	**80%**	**80%**
**Vascular (N = 27)**	16.2 ± 25.2	**70%**	**59%**
**Overall**	12.2 ± 20.7	**72%**	**72%**

**Table 6:** Recall and precision of random forest model predicting aggregate opioid requirements. Prediction results prior to surgery are shown.

Recall			Precision		
None + Low	Low + Medium	Medium + High	None + Low	Low + Medium	Medium + High
**88%**	**5%**	**41%**	**72%**	**50%**	**73%**
**(N = 1699)**	(N = 945)	(N = 1041)	(N = 1699)	(N = 945)	(N = 1041)

**Table 7:** Confusion matrix for each category of opioid requirement with true positive, true negative, false positive and false negative counts are shown. Also, presented are the True Positive Rate (TPR), False Negative Rate (FNR), False Positive Rate (FPR) and True Negative Rate (TNR). Positive Predictive Value (PPV), False Omission Rate (FOR), False Discovery Rate (FDR) and Negative Predictive Value (NPV) are also presented.

**Opioid category**		**Predicted**						
**None + Low**		**Positive**	**Negative**	**Total**				
**Actual**	**Positive**	1500	199	1699	TPR	88%	FNR	12%
	**Negative**	572	469	1041	FPR	55%	TNR	45%
	**Total**	2072	668	2740				
		PPV	FOR					
		72%	30%					
		FDR	NPV					
		28%	70%					
**Opioid category**		**Predicted**						
**Low + Medium**		**Positive**	**Negative**	**Total**				
**Actual**	**Positive**	43	902	945	TPR	5%	FNR	95%
	**Negative**	43	1752	1795	FPR	2%	TNR	98%
	**Total**	86	2654	2740				
		PPV	FOR					
		50%	34%					
		FDR	NPV					
		50%	66%					
**Opioid category**		**Predicted**						
**Medium + High**		**Positive**	**Negative**	**Total**				
	**Positive**	423	618	1041	TPR	41%	FNR	59%
	**Negative**	159	1540	1699	FPR	9%	TNR	91%
	**Total**	582	2158	2740				
		PPV	FOR					
		73%	29%					
		FDR	NPV					
		27%	71%					

### Feature importance

Feature importance of the Random Forest model, determined through permutation method, is presented in Table 8. The average relative importance of different features contributing to model prediction is outlined. The type of procedure, patient’s medical history and procedure duration were the top three features contributing to model predictions. Overall, patient features contributed 65% while procedure features contributed 35% towards model predictions.

**Table 8:** Feature importance of Random Forest Model explaining the relative importance of various features contributing to predictions of opioid requirements. Similar features are consolidated.

Features	Relative importance
Procedure type	**15.4%**
Medical History (Cardiac/Pulmonary/Neurological/Hepatic/Endocrine/Musculoskeletal/Renal/Cancer)	**12.9%**
Procedure Duration	**12.0%**
Age	**9.8%**
Surgical specialty	**8.3%**
Body Mass Index	**8.2%**
Home and preoperative pain medications (Opioid/Non opioid)	**7.3%**
Preoperative Pain Levels	**4.8%**
ASA Physical Status	**4.2%**
Race	**4.2%**
Social History (Tobacco/Alcohol/Recreational Drug Use)	**4.1%**
Psychiatric/Neurological issues (Anxiety/Depression/PTSD/Spinal Cord Injury)	**4.1%**
History or risk for sleep apnea	**2.1%**
Gender	**1.9%**
History of Chronic Pain	**0.6%**

## 4. Discussion

Management of acute postoperative pain with opioids needs to be optimal to avoid the adverse effects of overdose and underdose. Towards this, we applied artificial intelligence methods specifically, machine learning in this instance to predict postoperative opioid requirements so that proactive planning could be enabled. We used a comprehensive and large perioperative dataset to develop and validate the models which were trained to make predictions preoperatively prior to surgery and at the end of surgery. Our study showed that machine learning models can predict postoperative opioid requirements with an accuracy around 70% when adjacent opioid requirement categories are aggregated. Among the models tried, Random Forest, Multinomial regression and Extreme Gradient Boost models performed better than Naïve Bayes and Neural Network. The differences between the performances of these higher performing models were only marginal.

Several key findings are noted while observing the model predictions. Surprisingly, the model accuracies were very similar prior to surgery and at the end of surgery; suggesting that intraoperative data (intraoperative opioids, other types of analgesics, inhalation agents, fluids, patient position, etc.) did not contribute to improving model accuracy. This may prove to be advantageous because a model that can preoperatively predict the postoperative opioid requirements without compromising accuracy could potentially enable proactive pain management strategies prior to surgery.

Model sensitivity (recall) and precision were only modest at best and that too only for the “none+low” category. The model had particular difficulty predicting whether a patient’s opioid requirement would fall in the “low+medium” category with a tendency to misclassify the requirement as “none+low”. The model performance was better when predicting “none+low” as compared with other categories. This may explain why model accuracies varied for different surgical specialties. The specialties that had higher opioid requirements tended to have lower model accuracies.

Feature importance for best performing Random Forest model predictions made prior to surgery reveals interesting observations (Table 8). The scheduled procedure type proved to be the most important feature in model predictions. Yet, patient specific factors—demographics, medical history, social history, and psychiatric issues together played a predominant role in determining the postoperative opioid requirements.

Despite including a comprehensive dataset of preoperative and intraoperative parameters, model accuracies in predicting postoperative opioid requirements were not over 72%. This suggests that additional factors influencing opioid requirements were potentially not included in the data used for training the models. A potential factor that we considered was provider practice pattern in ordering opioids for pain management. However, adding surgeon and anesthesiologist data into the model led to no notable improvement. Accuracy of machine learning models can be, in principle, improved with more data. Additional data for model training could be obtained either by extending the time range of the dataset or obtaining data from more institutions. However, here are downsides to each approach. By extending the time range, the risk for encountering changes in practice and documentation patterns over time increases potentially compromising data consistency. Similarly, institutional variations in case mix and practices can negatively affect consistency of multi-institutional data. In this particular project, we noted that adding two additional years of data yielded no improvement in accuracy.

The single center nature of data is a limitation of the study and whether the model performance can be replicated in other centers is unknown at this time. As a future step training and validating the model against standardized multicenter data such as those hosted by Multicenter Perioperative Outcomes Group (www.mpog.org) could be a way to validate the model across institutions. The second limitation of this project is that we focused only on outpatients. This was deliberate to avoid the confounding factors of patient-controlled analgesia, regional blocks for postoperative pain management and variable length of stay that are difficult to incorporate into the model. For this reason, we chose to keep the scope limited to outpatients in this first modeling effort.

In summary, machine learning models were able to predict postoperative opioid requirements in ambulatory surgery patients. Prediction accuracies remained unchanged even after adding intraoperative information to preoperative data. In general, model prediction sensitivities were greater in patients requiring lower amounts of opioids as compared with those requiring higher amounts. Translating such models into point of care tools could provide assistive intelligence to the perioperative care provider leading to improved management of postoperative acute pain.

## Supporting information

S1 Table Oral Morphine Milligram Equivalents (MME) conversion ratios used for the study. (DOCX)

S2 Table Categorized procedure types used for modeling. (DOCX)

S3 Table Detailed prediction accuracies of random forest model for different specialties of surgeries when considering the single highest probability category. Prediction accuracies prior to and after surgery are shown.
(DOCX)

S4 Table Recall and precision of random forest model when considering the single highest probability category. Prediction recall and precision prior to surgery are shown.
(DOCX)
