# Supplementary material for: Machine learning approach to predict postoperative opioid requirements in ambulatory surgery patients
Source: PLoS One. 2020 Jul 31;15(7):e0236833. doi: 10.1371/journal.pone.0236833 (PMC7394436; doi:10.1371/journal.pone.0236833)
Supplement: S1 Table — (DOCX) [file pone.0236833.s001.docx]

**S1 Table:** Oral Morphine Milligram Equivalents (MME) conversion ratios used for the study.

| Name of Drug | Route | MME Conversion |
| --- | --- | --- |
| Morphine (mg) | IV | 3 |
| Fentanyl (mcg) | IV | 0.3 |
| Hydromorphone (mg) | IV | 20 |
| Codeine (mg) | IV | 0.25 |
| Methadone (mg) | IV | 3 |
| Buprenorphine (mg) | IV | 50 |
| Meperidine (mg) | IV | 0.4 |
| Oxycodone (mg) | Oral | 1.5 |
| Tramadol (mg) | Oral | 0.25 |
| Remifentanil (mcg) | IV | 0.45 |
| Alfentanil (mcg) | IV | 0.075 |
| Sufentanil (mcg) | IV | 3.0 |
