# Supplementary material for: Machine learning approach to predict postoperative opioid requirements in ambulatory surgery patients
Source: PLoS One. 2020 Jul 31;15(7):e0236833. doi: 10.1371/journal.pone.0236833 (PMC7394436; doi:10.1371/journal.pone.0236833)
Supplement: S2 Table — (DOCX) [file pone.0236833.s002.docx]

**S2 Table**: Categorized procedure types used for modeling

| Surgery Specialty | Procedure Type Description | Case Counts | Case Proportion (%) |
| --- | --- | --- | --- |
| General | Breast biopsy, lumpectomy, mastectomy | 953 | 7.2% |
| General | Local excision: head, neck, trunk | 486 | 3.7% |
| General | Laparoscopic: cholecystectomy, robot assisted | 173 | 1.3% |
| General | Laparoscopic: hernia repair, appendectomy | 219 | 1.6% |
| General | Laparoscopic: minor procedures, diagnostic | 205 | 1.5% |
| General | Laparoscopic: other surgeries | 42 | 0.3% |
| General | Open laparotomy, major | 30 | 0.2% |
| General | Open hernia repair; inguinal, umbilical, ventral | 130 | 1.0% |
| General | Lymph node biopsy, mapping | 93 | 0.7% |
| General | Hemorrhoidectomy | 79 | 0.6% |
| General | Trans-anal procedures | 258 | 1.9% |
| General | Exam under anesthesia | 132 | 1.0% |
| General | Parathyroidectomy, thyroidectomy | 153 | 1.2% |
| General | Other | 38 | 0.3% |
| Gynecology | Laparoscopic surgeries, Robot assist | 408 | 3.1% |
| Gynecology | Laparoscopic minor procedures, diagnostic | 374 | 2.8% |
| Gynecology | Hysteroscopy | 41 | 0.3% |
| Gynecology | Transvaginal procedures | 499 | 3.8% |
| Gynecology | Vulvectomy, local excision, repair | 30 | 0.2% |
| Gynecology | Other | 62 | 0.5% |
| Neuro | Muscle, nerve biopsy | 117 | 0.9% |
| Neuro | Pain pump, intrathecal pump, nerve stimulator | 337 | 2.5% |
| Neuro | Carpal tunnel release, trigger finger | 29 | 0.2% |
| Neuro | Minimally invasive discectomy | 126 | 0.9% |
| Neuro | Laminectomy, facetectomy, foraminotomy, discectomy | 65 | 0.5% |
| Neuro | Other | 25 | 0.2% |
| Oral | Dental extractions | 181 | 1.4% |
| Oral | Mandibular, maxillary, Temporomandibular joint | 49 | 0.4% |
| Oral | Other | 159 | 1.2% |
| Orthopedic | Shoulder arthroscopy | 165 | 1.2% |
| Orthopedic | Open: shoulder, sternal | 38 | 0.3% |
| Orthopedic | Upper extremities: clavicle, elbow, hand, finger, arthroscopy | 138 | 1.0% |
| Orthopedic | Upper extremities: soft tissue, biopsy, incision & drainage | 85 | 0.6% |
| Orthopedic | Hip arthroscopy | 63 | 0.5% |
| Orthopedic | Knee arthroscopy | 386 | 2.9% |
| Orthopedic | Open: knee | 30 | 0.2% |
| Orthopedic | Lower extremities; ankle, amputation, arthroscopy | 20 | 0.2% |
| Orthopedic | Lower extremities; soft tissue, biopsy, incision & drainage | 55 | 0.4% |
| Orthopedic | Other | 73 | 0.5% |
| Otolaryngology | Nasal: septoplasty | 757 | 5.7% |
| Otolaryngology | Endoscopic procedures | 208 | 1.6% |
| Otolaryngology | Ear: tympanoplasty, cochlear | 329 | 2.5% |
| Otolaryngology | Laryngoscopy, panendoscopy, vocal cord, CO2 laser | 571 | 4.3% |
| Otolaryngology | Excision, exploration, head, neck | 371 | 2.8% |
| Otolaryngology | Oral: tonsillectomy, glossectomy | 183 | 1.4% |
| Otolaryngology | Facial, eyelid, orbit | 46 | 0.3% |
| Otolaryngology | Other | 41 | 0.3% |
| Plastic | Excision: scalp, face, blepharoplasty | 136 | 1.0% |
| Plastic | Excision: neck, chest, mammoplasty | 1342 | 10.1% |
| Plastic | Excision: abdomen | 86 | 0.6% |
| Plastic | Excision: extremities | 55 | 0.4% |
| Thoracic | Endobronchial ultrasound, bronchoscopy | 219 | 1.6% |
| Thoracic | Bronchoscopy procedures, trachea | 146 | 1.1% |
| Thoracic | Mediastinoscopy | 61 | 0.5% |
| Thoracic | Esophagus | 20 | 0.2% |
| Vascular | Arteriovenous fistula | 76 | 0.6% |
| Vascular | Lower extremities | 78 | 0.6% |
| Vascular | Other | 13 | 0.1% |
| Urology | Cystoscopy, biopsy, stent, prostate seed | 1135 | 8.5% |
| Urology | Transurethral procedures | 404 | 3.0% |
| Urology | Lithotripsy | 66 | 0.5% |
| Urology | Vasectomy, hydrocelectomy, orchiectomy, sacral | 145 | 1.1% |
| Urology | Penile | 145 | 1.1% |
| Urology | Other | 47 | 0.4% |
| Other | Bone marrow | 77 | 0.6% |
