# Supplementary material for: Machine learning approach to predict postoperative opioid requirements in ambulatory surgery patients
Source: PLoS One. 2020 Jul 31;15(7):e0236833. doi: 10.1371/journal.pone.0236833 (PMC7394436; doi:10.1371/journal.pone.0236833)
Supplement: S3 Table — Prediction accuracies prior to and after surgery are shown. (DOCX) [file pone.0236833.s003.docx]

**S3 Table:** Detailed prediction accuracies of random forest model for different specialties of surgeries when considering the single highest probability category. Prediction accuracies prior to and after surgery are shown.

| Surgical Specialty | Mean opioid requirement (MME) | Accuracy | |
| --- | --- | --- | --- |
|  |  | Beginning of surgery | End of surgery |
| General  (N=603) | 12.1 ± 17.8 | **46%** | **48%** |
| Gynecology  (N=289) | 11.0 ± 17.2 | **48%** | **48%** |
| Neuro  (N=132) | 12.6 ± 25.1 | **51%** | **52%** |
| Oral  (N=94) | 7.7 ± 17.1 | **71%** | **74%** |
| Orthopedic  (N=219) | 19.7 ± 21.2 | **41%** | **47%** |
| Otolaryngology  (N=495) | 11.3 ± 25.4 | **45%** | **45%** |
| Plastic  (N=317) | 20.0 ± 23.4 | **40%** | **40%** |
| Thoracic  (N=96) | 2.9 ± 8.4 | **89%** | **89%** |
| Urology  (N=393) | 6.8 ± 14.4 | **70%** | **70%** |
| Vascular  (N=27) | 16.2 ± 25.2 | **44%** | **37%** |
| Overall | 12.2 ± 20.7 | **51%** | **52%** |
