# Supplementary material for: Machine learning approach to predict postoperative opioid requirements in ambulatory surgery patients
Source: PLoS One. 2020 Jul 31;15(7):e0236833. doi: 10.1371/journal.pone.0236833 (PMC7394436; doi:10.1371/journal.pone.0236833)
Supplement: S4 Table — Prediction recall and precision prior to surgery are shown. (DOCX) [file pone.0236833.s004.docx]

**S4 Table:** Recall and precision of random forest model when considering the single highest probability category. Prediction recall and precision prior to surgery are shown.

| Recall | | | | Precision | | | |
| --- | --- | --- | --- | --- | --- | --- | --- |
| None | Low | Medium | High | None | Low | Medium | High |
| 93%  (N=1290) | 2%  (N=409) | 14%  (N=536) | 26%  (N=505) | 55%  (N=1290) | 24%  (N=409) | 32%  (N=536) | 42%  (N=505) |
